# Validation of the PEDiatric Behçet’s Disease classification criteria: an evidence-based approach

**DOI:** 10.1093/rheumatology/kead609

**Published:** 2023-11-22

**Authors:** Caterina Matucci-Cerinic, Helene Palluy, Sulaiman M Al-Mayouf, Paul A Brogan, Luca Cantarini, Ahmet Gul, Ozgur Kasapcopur, Jasmin Kuemmerle-Deschner, Seza Ozen, David Saadoun, Farhad Shahram, Francesca Bovis, Eugenia Mosci, Nicolino Ruperto, Marco Gattorno, Isabelle Kone-Paut

**Affiliations:** UOC Rheumatology and Autoinflammatory Diseases, IRCCS Istituto G. Gaslini, Genoa, Italy; Department of Neurosciences, Rehabilitation, Ophthalmology, Genetics, Maternal and Child Health (DINOGMI), University of Genoa, Genoa, Italy; Pediatric Rheumatology and CEREMAIA, Bicêtre Hospital, APHP, University Paris Saclay, Paris, France; Department of Pediatrics, King Faisal Specialist Hospital and Research Center, College of Medicine, Alfaisal University, Riyadh, Saudi Arabia; University College London, Great Ormond Street Institute of Child Health, London, UK; Rheumatology Unit, Department of Medical Sciences, Surgery and Neurosciences, University of Siena, Siena, Italy; Division of Rheumatology, Department of Internal Medicine; Istanbul Faculty of Medicine, Istanbul University, Istanbul, Turkey; Department of Pediatric Rheumatology, Cerrahpasa Medical School, Istanbul University – Cerrahpasa, Ankara, Turkey; Division of Pediatric Rheumatology, Department of Pediatrics and autoinflammation reference center Tuebingen, University Hospital Tuebingen, Tuebingen, Germany; Department of Pediatric Rheumatology, Hacettepe University, Ankara, Turkey; Department of Internal Medicine and Clinical Immunology and CEREMAIA, AP-HP Groupe Hospitalier Pitié-Salpêtrière, Sorbonne University, Paris, France; Rheumatology Research Center, Shariati Hospital, Tehran University of Medical Sciences, Tehran, Iran; Department of Health Sciences (DISSAL), University of Genoa, Genoa, Italy; Gaslini Trial Center, IRCCS Istituto Giannina Gaslini, Genova, Italy; Gaslini Trial Center, IRCCS Istituto Giannina Gaslini, Genova, Italy; UOC Rheumatology and Autoinflammatory Diseases, IRCCS Istituto G. Gaslini, Genoa, Italy; Pediatric Rheumatology and CEREMAIA, Bicêtre Hospital, APHP, University Paris Saclay, Paris, France

**Keywords:** Behçet’s syndrome, classification criteria, vasculitis, pediatric, PEDBD

## Abstract

**Objectives:**

To validate the PEDiatric Behçet’s Disease classification criteria (PEDBD) with an evidence-based approach.

**Methods:**

A total of 210 pediatric patients [70 Behçet’s disease (BD), 40 periodic fever, aphthous stomatitis, pharyngitis, adenitis, 35 familial Mediterranean fever, 26 hyper-IgD syndrome, 22 TNF-receptor associated periodic fever syndrome, 17 undefined recurrent fevers] were randomly selected from the Eurofever Registry. A set of 11 experienced clinicians/researchers blinded to the original diagnosis evaluated the patients. Using the table consensus as gold standard (agreement ≥ 80%), the PEDBD, ISG and ICBD criteria were applied to BD patients and to confounding diseases with other autoinflammatory conditions in order to define their sensitivity, specificity and accuracy.

**Results:**

At the end of the third round, a consensus was reached in 139/210 patients (66.2%). The patients with a consensus ≥80% were classified as confirmed BD (*n* = 24), and those with an agreement of 60–79% as probable BD (*n* = 10). When comparing these patients with the confounding diseases group, an older age at disease onset, the presence of oral and genital ulcers, skin papulo-pustular lesions, a positive pathergy test and posterior uveitis were BD distinctive elements. The ISG, ICBD and PEDBD criteria were applied to confirmed BD and to the confounding disease group, showing a sensitivity of 0.50, 0.79 and 0.58, a specificity of 1.00, 0.97 and 0.99, and an accuracy of 0.91, 0.94 and 0.92, respectively.

**Conclusions:**

The PEDBD criteria were very specific, while the ICBD were more sensitive. The complexity of childhood BD suggests larger prospective international cohorts to further evaluate the performance of the criteria.

## Graphical abstract

**Figure:**
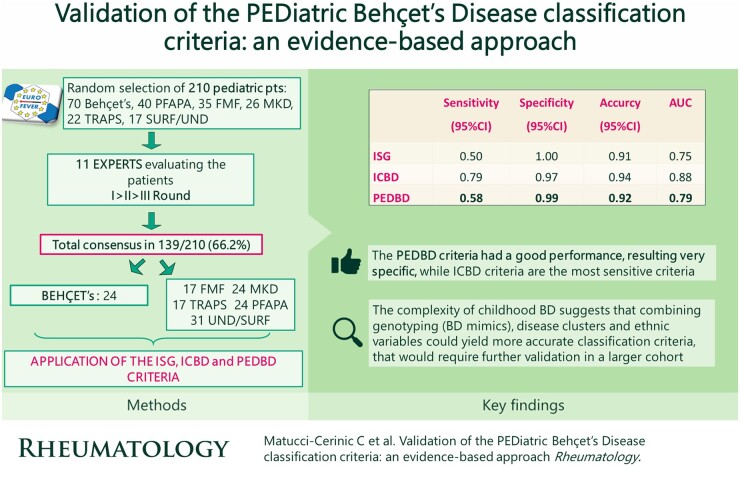

Rheumatology key messages

- Oral, genital and perianal ulcers, posterior uveitis, and positive pathergy test are pediatricBD distinctive features.
- PEDBD criteria are very specific in classifying pediatric Behçet’s disease.

## Introduction

Behçet’s disease (BD), is a systemic inflammatory multiorgan disease displaying features of both autoinflammation and vasculitis [1, 2]. Classified as a ‘variable vessel vasculitis’ [3], the disease is characterized by an involvement of the skin, eyes, joints, gut and central nervous system. The geographical distribution of BD shows a prevalence along the eastern and Mediterranean countries, and its phenotypic expression varies among different populations. The disease pathogenesis still remains unclear, involving both the innate and T cells-mediated immunity, with MHC I-associated predisposing factors [4].

In children, BD is rare and its diagnosis challenging due to a gap between the onset of the first manifestations and the development of a complete clinical picture. In fact, the first BD symptoms may start in early childhood, reaching a complete form before the age of 16 years in 4–26% of cases only [5, 6].

The variability of the disease features (age, ethnic background, disease clusters) has generated several sets of diagnostic and classification criteria, making BD the vasculitis with the highest number of criteria ever created (18 sets). In 1946, the first diagnostic criteria were published [7], followed in the next decades by several sets of criteria, highlighting also the geographical differences in the clinical presentation of BD. In the 1990s, the first international criteria were proposed (ISG) [8], then revised in 2006 [9] and 2013 (ICBD) [10]. Because pediatric BD was not addressed specifically in any of the previous criteria, in 2010 an international prospective cohort of pediatric BD was created, leading to the first set of pediatric BD classification criteria in 2015 (PEDBD) [5, 11]. These criteria were tested in some pediatric cohorts with different results in sensitivity and specificity [12–17].

Initially, ISG were defined ‘diagnostic’, but in 1989 (V International Conference on BD) they were proposed as ‘classification’ criteria, because they were considered more ‘useful in ensuring the uniformity of groups of patients for clinical and laboratory studies and for teaching purposes’ [18]. Diagnostic criteria are a set of signs, symptoms and tests used in order to make the diagnosis, and are consequently broad, so as to represent the disease heterogeneity, and to identify a large number of patients with the disease (high sensitivity). On the other hand, classification criteria are standardized definitions used to identify homogeneous cohorts for clinical studies, selecting the majority of patients with characteristic disease features and not the entire group of patients with the diagnosis (high specificity) [19].

In pediatrics, BD presentation is heterogeneous and the differential diagnosis challenging. Therefore, highly specific classification criteria are mandatory to stratify patients for clinical trials and research purposes.

The aim of the present study was to evaluate the degree of consensus on BD classification in patients enrolled in a large international registry, and to evaluate the performance of the ISG, ICBD and PEDBD classification criteria in a cohort of internationally validated pediatric BD, through an international clinicians/researchers (expert)-based consensus process.

## Methods

Data were extracted from the Eurofever Registry [20], whose main characteristics, diseases involved and selection of variables were already described [20, 21]. The Ethics Committe of Gaslini Insitute approved the Eurofever Registry on 18 June 2009. Three further amendments have received the favourable opinion of the Ethics Committee of Regione Liguria on 17 March 2015, 26 October 2020 and 13 December 2021. Informed consents or assents were obtained from parents, or patients, as appropriate in each of the participating centres, according to local regulations. Ethics committee approval has been obtained by the participating centres according to local or national regulations.

For the purpose of this study, 70 patients with a BD diagnosis formulated by the physician taking care of the patients, were randomly selected, and matched to 140 patients as controls, chosen among autoinflammatory diseases (familial Mediterranean fever, FMF; mevalonate kinase deficiency, MKD; TNF-receptor associated periodic fever syndrome, TRAPS; periodic fever, aphthous stomatitis, pharyngitis, adenitis, PFAPA; undefined inflammatory syndromes/syndrome of undifferentiated recurrent fevers, UND/SURF).

Inclusion criteria were the onset of the first symptom before 16 years of age, and the presence of the information required for the application of the different sets of BD criteria (data – Yes/No – about muco-cutaneous, neurological, ocular and vascular manifestations mandatory). The patients previously included in the creation of the 2015 PEDBD criteria were excluded. For confounding diseases, the same inclusion criteria were applied. Genetic testing information were also included for FMF, MKD and TRAPS, when available.

Eleven international clinicians experienced in BD and autoinflammatory diseases (four adult and seven pediatric rheumatologists), blinded to patients’ original diagnosis, participated in a multi-round robin secured web process according to the Nominal Group Technique (NGT) [22, 23] to classify each of the 210 patients into one of the six mutually exclusive diseases (BD, PFAPA, FMF, MKD, TRAPS, SURF/UND). The experts evaluated patients’ data describing clinical manifestations from disease onset to the Eurofever enrolment visit. In the first round, only clinical and laboratory data were evaluated; in the second round, data about genetic analysis were added; in the third round, other experts’ comments were shown. A consensus was considered for an agreement of at least 9/11 experts (82%). For BD, patients with a consensus (≥80%) were considered as ‘confirmed BD’, those with an agreement in 7–8/11 experts (60–79%) as ‘probable BD’, while patients with an agreement inferior to 60% were considered as ‘uncertain BD’.

Three different sets of criteria: the ISG [8], the revised-ICBD [10] and the PEDBD [5] (Supplementary Table S1, available at *Rheumatology* online) were then tested in confirmed BD, probable BD and in the confounding diseases with a consensus.

### Statistical analysis

Descriptive statistics are reported as medians [first to third quartile] for continuous variables, and as absolute frequencies and percentages for categorical variables. Comparisons of disease characteristics between patients’ groups were performed by χ^2^ test or Kruskall–Wallis test, as appropriate.

The three sets of criteria (ISG, ICBD, PEDBD) were applied to confirmed and probable BD. The confounding diseases with a consensus >80% served as controls. For each criterion, sensitivity, specificity, accuracy and area under the ROC curve (AUC) were calculated.

## Results

### Consensus process

**Figure 1.**
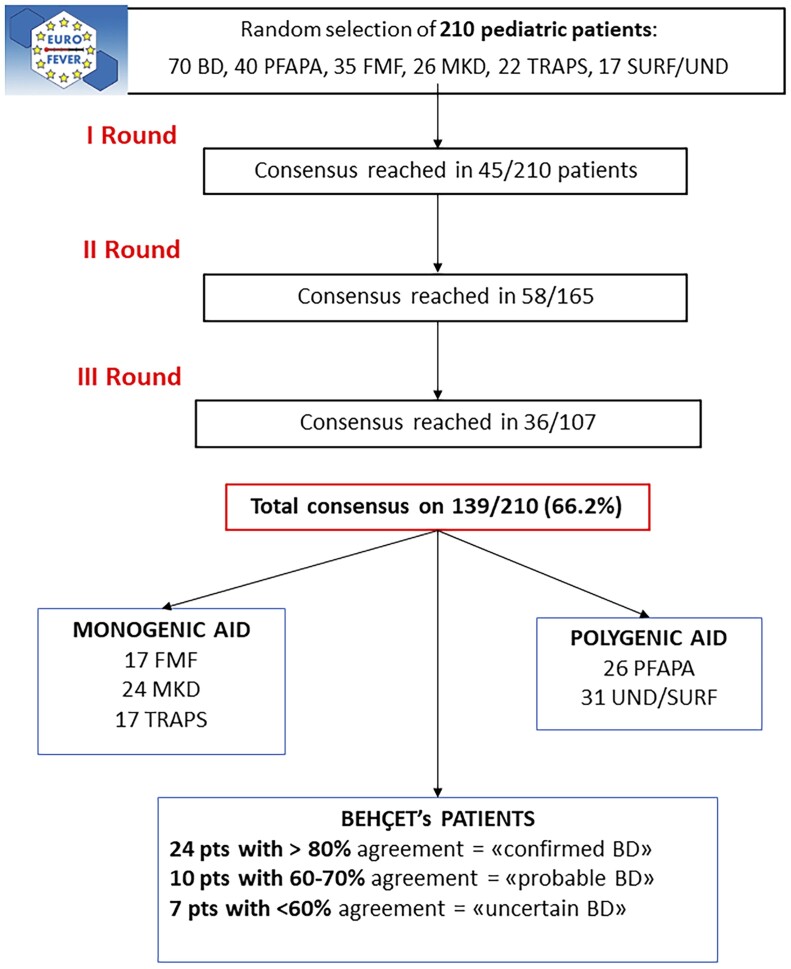
Flowchart of the evaluation process

The data from the first visit of 210 patients—70 patients with BD and 140 patients with confounding diseases (40 PFAPA, 35 FMF, 26 MKD, 22 TRAPS, 17 UND/SURF)—were randomly selected from the Eurofever registry. Adjudication of cases required three web-based consensus rounds. In the first round, a consensus (>80%) was obtained for 45, in the second round for 58, and in the third round for 36 patients, for a total consensus in 139/210 patients (66.2%). In Fig. 1 the evaluation process is shown, while in Supplementary Fig. S1 (available at *Rheumatology* online) all the votes per each disease are reported.

#### Consensus in BD patients

After the evaluation, 24 BD patients were defined as ‘confirmed BD’ (consensus ≥80%), 10 patients as ‘probable BD’ (consensus 60–79%) and seven patients as ‘uncertain BD’ (six patients having a consensus of 54% and one patient of 45%). Out of the initial 70 BD patients, 15 were classified as UND/SURF and two as PFAPA. In the seven ‘uncertain’ BD patients, the experts were divided between BD and UND/SURF.

#### Consensus in the confounding diseases

A final consensus was reached in 115/140 patients with confounding diseases. For MKD, a consensus was reached in 24 patients [23/26 initially enrolled as MKD (88.4%), plus one initially enrolled as PFAPA]. The three MKD with a partial agreement showed MVK gene heterozygous pathogenic mutations, and one likely pathogenic mutation. In TRAPS, a consensus was reached in 17/22 patients (77.3%): all the five patients with a partial/absent agreement presented the low significance variant R92Q in the *TNFRSF1A* gene. In FMF, 19/35 patients (54.3%) reached a consensus, whereas nine patients reached only a partial agreement (all of them presented heterozygous pathogenic *MEFV* mutations). A final consensus on PFAPA was achieved in 26 patients [22/40 of the initial PFAPA cohort (55%), two patients initially enrolled as FMF and two as UND/SURF]. For UND/SURF a consensus was achieved in 31 patients: [9/17 initial UND/SURF (53%), five initially enrolled as PFAPA, two as FMF and 15 as BD] (Supplementary Fig. S1, available at *Rheumatology* online).

### Demographic and clinical data

The demographic characteristics of the whole initial cohort are reported in Supplementary Table S2 (available at *Rheumatology* online), while in Table 1 the demographic characteristics of the consensus cohort are reported.

**Table 1.** Demographic characteristics of confirmed BD, probable BD and of the confounding diseases with consensus

	C-BD	P-BD	TRAPS	MKD	FMF	PFAPA	UND/SURF
	N = 24	N = 10	N = 17	N = 24	N = 17	N = 26	N = 31
Male gender, *n* (%)	16 (67%)	8 (80%)	12 (71%)	12 (50%)	10 (59%)	12 (46%)	13 (42%)
Age at onset (years) median [first to third quartiles]	7.95 [6.59–11.72]	10.43 [6.55–11.97]	1.08 [0.11–3.78]	0.29 [0.11–0.54]	3.73 [1.89–5.11]	1.41 [0.85–2.56]	3.83 [1.60–6.49]
Age at diagnosis (years) median [first to third quartiles]	11.25 [8.48–12.31]	11.85 [9.98–13.91]	6.75 [2.83–8.73]	4.41 [2.17–5.81]	6.12 [4.54–9.57]	3.46 [2.23–5.63]	8.44 [4.31–11.02]
Disease duration between disease onset and first Eurofever visit (years) median [first to third quartiles]	1.23 [0.57–3.88]	1.85 [0.96–2.51]	6.50 [0.57–2.0–8.24]	5.91 [1.67–8.56]	3.62 [2.1–9.59]	4.28 [3.19–8.5]	3.63 [1.38–6.44]
Ethnicity, *n* (%)							
Caucasian (European)	23 (96%)	9 (90%)	16 (94%)	22 (96%)	12 (71%)	25 (95%)	30 (97%)
Asian	1 (4%)	0	0	0	0	1 (5%)	0
Middle-East	0	0	1 (6%)	1 (4%)	3 (18%)	0	0
Other	0	1 (10%)	0	0	2 (12%)	0	1 (3%)
Affected relatives, *n* (%)	4/23 (17%)	1 (10%)	10 (59%)	4/22 (18%)	8 (47%)	3 (14%)	8 (26%)
Consanguinity, *n* (%)	1 (4%)	0	1 (6%)	0	1 (6%)	0	1 (3%)

C-BD: confirmed Behçet’s Disease; FMF: familial Mediterranean fever; MKD: mevalonate kinase deficiency; P-BD: probable Behçet’s Disease; PFAPA: periodic fever, aphthous stomatitis, pharyngitis, adenitis; TRAPS: TNF-receptor associated periodic fever syndrome; UND/SURF: undefined inflammatory syndromes/syndrome of undifferentiated recurrent fevers.

Patients were prevalently Caucasian, and the age at disease onset varied among the different populations, with a lower age at first symptom for FMF, MKD and UND/SURF.

#### BD cohort

In confirmed BD patients, a positive family history was present in 17% of patients, and the age onset (median) was 7.95 years with an earlier appearance of symptoms in females (mean 7.5 years in females *vs* 9.2 years in males, see Table 1). Oral ulcers (OU) were the clinical sign shared by all patients, 77% had genital ulcers (GU), 35% pseudo-folliculitis, 32% papulo-pustular lesions or acne and 8% erythema nodosum. In 39% of patients, pathergy test was positive, being a relevant sign distinguishing BD from confounding diseases (in this category, data on skin pathergy reactions were sometimes missing). In 54% of patients, ocular manifestations were found: anterior (29%) and posterior uveitis (27%), retinal vasculitis and papillary oedema (8%), and impaired vision (17%). Vascular involvement was rare and limited to venous thrombosis (8%). Neurologic manifestations were present in 42% of patients: 25% had an isolated headache (the most frequent symptom), and 17% cranial nerve palsy. In 38% of patients GI symptoms were present, mainly as abdominal pain (33%), diarrhea and gastrointestinal bleeding (13%) and anal/perianal ulcers (8%). In 33% of patients, musculoskeletal manifestations were detected (29% arthralgia and 13% arthritis). Fever was present in 50% of patients, with a prevalently irregular pattern. HLA-B51 was present in 69% of patients (See Table 2 and Supplementary Table S3, available at *Rheumatology* online).

**Table 2.** Clinical manifestations in confirmed and probable BD

	C-BD	P-BD
	N = 24	N = 10
Aphtous stomatitis, *n* (%)	24 (100%)	10 (100%)
Genital ulcers, *n* (%)	17/22 (77%)	4 (40%)
Skin manifestations, *n* (%)	12 (50%)	7 (70%)
Positive pathergy test, *n* (%)	7/18 (39%)	1 (10%)
Ocular manifestations, *n* (%)	13 (54%)	2 (20%)
Venous thrombosis, *n* (%)	2 (8%)	0
Neurologic manifestations, *n* (%)	10 (42%)	1 (10%)
Gastrointestinal manifestations, *n* (%)	9 (38%)	3 (30%)
Musculo-skeletal manifestation, *n* (%)	8 (33%)	4 (40%)
Fever, *n* (%)	12 (50%)	2 (20%)
HLA-B51, *n* (%)	11/16 (69%)	5/7 (71%)

C-BD: confirmed Behçet’s Disease; P-BD: probable Behçet’s disease.

**Figure 2.**
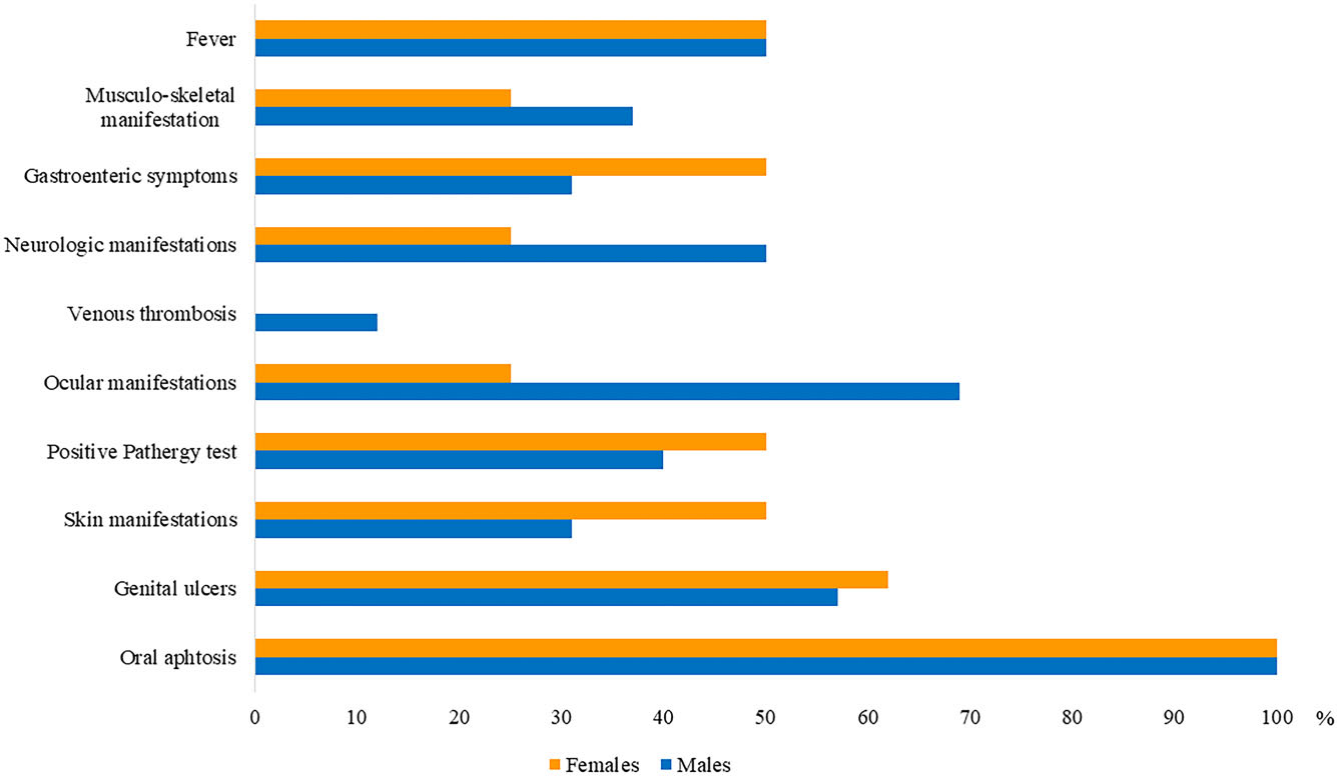
Frequency of the clinical manifestations in male and female confirmed BD patients

Females more frequently presented GU, skin manifestations and gastroenteric symptoms. On the contrary, ocular, vascular and neurologic involvement occurred more frequently in males. The general frequency of the different manifestations in the two genders is reported in Fig. 2, while in Supplementary Fig. S2 (available at *Rheumatology* online) the frequency of each manifestation per gender is summarised.

Among patients with probable BD, four had oral and genital aphthous ulcers and one had an anterior uveitis associated with oral aphthosis. The others were assigned to this group because of unusual features such as maculopapular rash, early age onset and in the absence of enough typical manifestations necessary to make a diagnosis of BD.

#### Comparison between confirmed BD—probable BD—uncertain BD, and initial BD subsequently classified as other diseases

The clinical characteristics of these groups are reported in Supplementary Table S3, available at *Rheumatology* online. In confirmed BD, the most frequent symptoms were GU (77%), positive pathergy test (39%) and posterior uveitis (27%) when compared with the other cohorts. A significant statistical difference (*P* <0.05) was found between these groups for GU, posterior uveitis, fever, migrant rash, palpable purpura, erythematous plaques and parotitis.

#### Confounding diseases cohort

The clinical characteristics of the whole cohort compared with BD are listed in Supplementary Table S4 (available at *Rheumatology* online), while the genetic characteristics for monogenic and polygenic autoinflammatory disease are reported, respectively, in Supplementary Tables S5 and S6 (available at Rheumatology online).

#### Comparison of BD cohort and confounding diseases cohort

**Figure 3.**
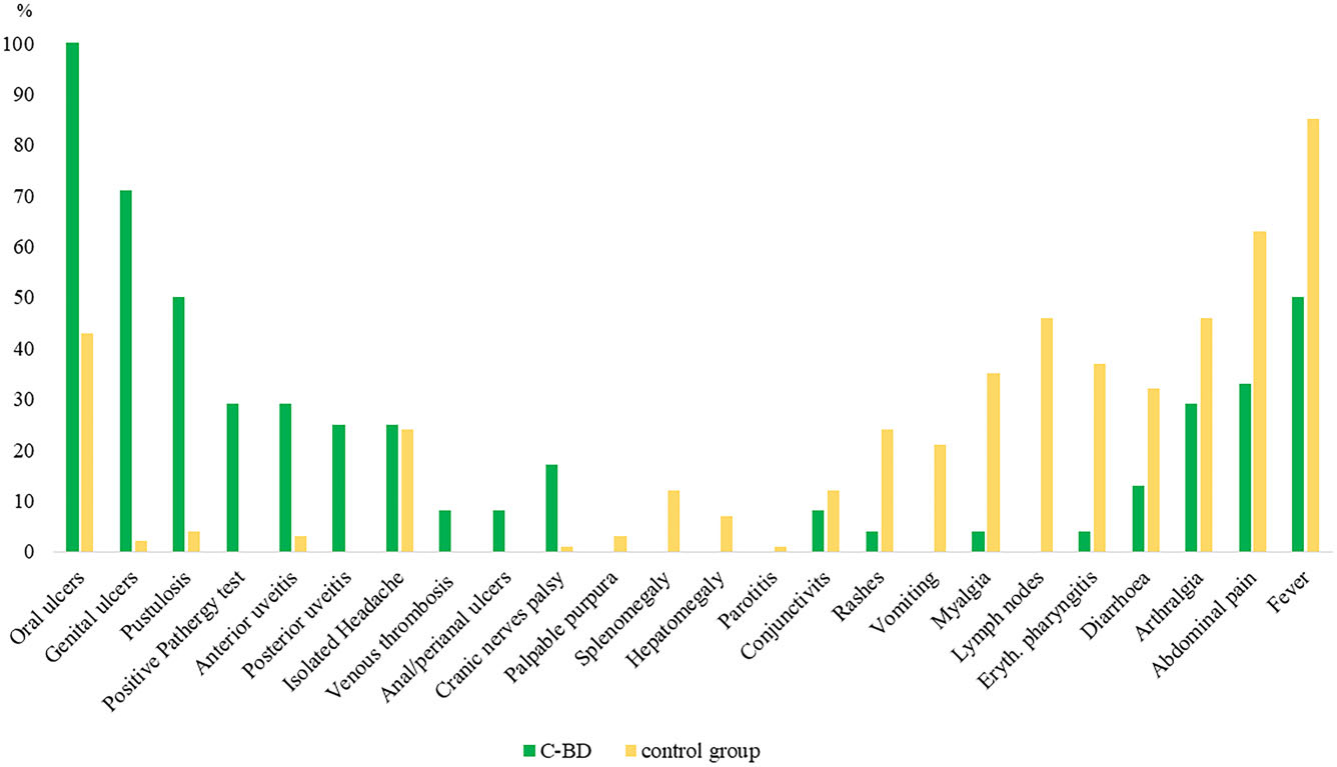
Clinical manifestations in the BD cohort and confounding diseases

The confirmed BD and probable BD patients presented a later disease onset and a shorter disease duration at the time of the first Eurofever visit when compared with confounding diseases (see Table 1). Monogenic diseases (especially TRAPS and FMF) had a more frequent family history of the disease. OU, GU and posterior uveitis were most frequently associated to BD, perianal ulcers were present only in BD patients (8%) and pseudofolliculitis, papulo-pustular lesions and acne were more frequent in BD, while skin rashes (macular, urticarial, erysipelas) were more frequently reported in other cohorts (Fig. 3). The statistical analysis showed a significant difference (*P* <0.001) between the confirmed BD and the confounding diseases for: age at disease onset and at diagnosis, family history, skin manifestations (OU, GU, acne, PT), anterior and posterior uveitis, myalgia, laterocervical lymphoadenopathies, chest pain, GI symptoms (vomiting, diarrhea, abdominal pain), pharyngitis and fever. For generalized lymphadenopathy, hepatosplenomegaly, pleurisy, papulo-pustular lesions, conjunctivitis, periorbital pain, macular rash, arthralgia, cranial nerve palsy and low fever a *P* <0.05 was found (Supplementary Table S4, available at *Rheumatology* online).

### Performance of the ISG, ICBD and PEDBD criteria

The three sets of criteria were applied to confirmed BD, probable BD and to confounding diseases with a consensus, to calculate their sensitivity, specificity and accuracy.

Of the 24 confirmed BD, 50% of patients fulfilled the ISG, 79.2% the ICBD and 58.3% the PEDBD criteria. In probable BD patients, only 1/10 (10%) fulfilled the ISG and PEDBD criteria, while 5/10 (50%) fulfilled the ICBD criteria. The major fulfilment of these latter criteria was due in four cases to bipolar aphthosis (OU+GU) and in one case to anterior uveitis together with OU. In the confounding diseases group, none fulfilled the ISG criteria, three fulfilled the ICBD criteria (two MKD and one UND/SURF) and one MKD fulfilled the PEDBD criteria (see Supplementary Table S7, available at *Rheumatology* online).

In Table 3, the performance of the three sets of criteria in confirmed and probable BD is reported.

**Table 3.** Sensitivity, specificity, accuracy and area under the ROC curve (AUC) of the three sets of criteria in 24 confirmed Behçet’s disease and 115 controls

24 CONFIRMED-BD *vs* 115 controls				
	Sensitivity (95%CI)	Specificity (95%CI)	Accuracy (95%CI)	AUC (95%CI)
ISG	0.50 (0.29–0.71)	1.00 (0.97–1.00)	0.91 (0.85–0.95)	0.75 (0.67–0.82)
ICBD	0.79 (0.58–0.93)	0.97 (0.92–0.99)	0.94 (0.89–0.97)	0.88 (0.82–0.93)
PEDBD	0.58 (0.37–0.78)	0.99 (0.95–1.00)	0.92 (0.86–0.96)	0.79 (0.71–0.85)

10 PROBABLE-BD *vs* 115 controls				
	Sensitivity (95%CI)	Specificity (95%CI)	Accuracy (95%CI)	AUC (95%CI)
ISG	0.10 (0.03–0.44)	1.00 (0.97–1.00)	0.93 (0.86–0.97)	0.55 (0.46–0.64)
ICBD	0.40 (0.12–0.74)	0.97 (0.92–0.99)	0.93 (0.86–0.97)	0.69 (0.60–0.77)
PEDBD	0.10 (0.03–0.45)	0.99 (0.95–1.00)	0.92 (0.85–0.96)	0.55 (0.45–0.64)

BD: Behçet’s disease; ISG: International Study Group classification criteria [8]; ICBD: revised International Criteria for Behçet's Disease [10]; PEDBD PEDiatric Behçet’s Disease criteria [5].

In Supplementary Fig. S3 (available at *Rheumatology* online), the percentage of patients in BD and control group presenting the manifestations required for the PEDBD criteria is shown: 100% presented OU, 77% GU, 50% typical skin manifestations (folliculitis, erythema nodosum, acne), 42% ocular disease (anterior/posterior uveitis or retinal vasculitis), 21% neurological manifestations (not including isolated headache) and 8% a vascular disease. These manifestations were present also in part of the probable BD patients (100% OU, 40% GU, 50% skin, 10% ocular), while they were, with the exception of OU, almost absent in the confounding diseases.

## Discussion

In children, BD is rare and difficult to recognize. The range of possible presentations and ages of onset reflects a clinical heterogeneity underpinned by different ethnic and genetic background. In pediatric age, the definition of BD represents an unmet need, because dedicated research studies and validated therapeutic trials are lacking. The PEDBD classification criteria were the first attempt to accurately classify BD children. Since then, few studies evaluated these criteria with different results, due to the lack of a gold-standard for diagnosis. To overcome this problem, common to many rheumatic diseases [23–29], an adequate validation process using surrogate standards such as expert consensus and comparison cohorts serving as controls, is essential. Therefore, to validate the PEDBD classification criteria, an international experienced clinicians/researchers consensus multi-round approach was used to evaluate their performance in a cohort of BD patients and of control diseases extracted from the Eurofever registry.

The experts evaluated 210 patients, with a consensus found in 66.2% of patients. During the patients’ classification process, it was found that the majority of patients without a consensus belonged to the BD and PFAPA groups, finally classified by the experts as UND/SURF. At the end of the consensus, while 70 patients were part of the initial BD EUROFEVER cohort, one third (*n* = 24) were classified as confirmed BD and 10 as probable BD. This result indicates that a probable over-diagnosis of BD in pediatrics is made, with a frequent confusion with other more common inflammatory pediatric conditions.

In line with other pediatric and adult cohorts [12, 16, 30–35], male patients presented a prevalent ocular, vascular and neurological involvement, while females presented more GU and a prevalent GI involvement. Skin manifestations (acne, papulo-pustular lesions, pseudo folliculitis) were almost equal in the two genders, while EN was more frequent in males. In adults, skin manifestations are associated with male gender [31–35], whereas our findings are in agreement with the pediatric literature [5], where skin manifestations are almost equal in both sexes. This could be due to a lack of pubertal hormones that could explain a different disease expression in children and adult patients.

Our study, comparing patients with confirmed BD with other confounding diseases, demonstrated that oral ulcers, genital ulcers, a positive pathergy test and the presence of posterior uveitis were distinctive BD features. Interestingly, in half of confirmed BD patients a history of recurrent fever was present, similarly to other pediatric cohorts [5, 12, 15]. In adulthood, fever is reported in 20% of cases [36]. However, the high prevalence of fever in BD children, independently from any vascular complication, raises several hypotheses. First, some patients could have an initial disease presentation with recurrent fevers, similar to PFAPA [37]; second, fever could be a clue for monogenic Behçet-like diseases, like A20 haploinsufficiency; third, fever could be a more frequent symptom in childhood, given the higher number of infectious episodes that could act as disease triggers.

The validated cohort of patients was used for the application of three sets of BD criteria: ISG, revised ICBD and PEDBD criteria, allowing the calculation of sensitivity, specificity and accuracy of each set of criteria. The ISG criteria require oral aphthosis as a mandatory criterion, associated with at least two other criteria (cutaneous, ocular, pathergy test). Their sensitivity in the confirmed BD patients was of 50%, with a very high specificity (100%). The ICBD criteria are less strict, with weighted items that allow a classification with the presence of only two symptoms among oral ulcers, genital ulcers and uveitis, and include other BD features (neurological and vascular). In our cohort, the ICBD had the best performance, reaching a specificity of 97% and a sensitivity of 79%. Similar results have been reported in other pediatric cohorts [12, 13, 15], but with a lower sensitivity than in the adult literature [38] and in the studies of Shahram and Ekinci [14, 17]. The lower sensitivity in our cohort could be due to the fact that the experts ruled out patients presenting only two symptoms from the confirmed BD cohort (five patients in the probable BD cohort).

The sensitivity of the PEDBD criteria was 58%, and the specificity 99%. When compared with other cohorts, their sensitivity was higher than that reported by Gallizzi, Butbul Aviel and Shahram [12–14], but lower than that reported by Koné-Paut and Batu [5, 16]. The specificity instead was higher than in all other cohorts.

It is interesting to note that while all the presented criteria are extremely specific, they generally suffer from a lower sensitivity. This can be explained by the fact that BD has unique features that help differentiate it from controls, but often in childhood, due to the time taken for the development of a complete clinical picture, the patients do not immediately meet all the items required by the criteria. At the same time, classification criteria need to be extremely specific to avoid the inclusion of patients with different diseases in studies. Therefore, this study confirms the suitability of the PEDBD classification criteria.

In the present study, the ICBD criteria displayed a slightly lower specificity and better sensitivity compared with PEDBD. These criteria enable the classification of BD with the presence of only two symptoms between oral and genital aphthosis. According to many experts, this issue may increase the risk of an over-classification, particularly when considering other confounding diseases not addressed in the present study, such as IBD or monogenic Behçet-like disorders, frequently associated with complex aphthosis. Moreover, BD-associated oral and genital ulcers have very specific features that require an experienced clinician to differentiate them from the aphthous ulcers found in other diseases. For this reason, we suggest that the PEDBD criteria, which require at least three BD-associated features, should still be considered the most reliable tool for classifying BD in children; the comparatively lower sensitivity, however, emphasizes that these classification criteria should not be misused as diagnostic criteria in routine clinical practice.

This study has some limitations. The number of confirmed and probable BD patients was lower [34] than expected when the initial 70 patients were randomly chosen from Eurofever. The data presented to the experts included all the clinical variables observed from disease onset to the enrolment of the patients in the Registry. Despite the median disease duration being longstanding, it is conceivable that for many experts the overall number of the reported clinical variables was not enough to consider the patients as confirmed BD. This could be due to the fact that in pediatric BD, often several years may pass between the disease onset and the development of a complete BD phenotype. Our study has included for comparison a number of patients with monogenic and multifactorial autoinflammatory diseases. However, we have not included other confounding diseases such as A20 haplo-insufficiency or other forms of vasculitis. It is worth noting, in that context, that clear vasculitic manifestations were observed in only a limited percentage of our pediatric BD population. Furthermore, the most frequently observed vasculitides in children, IgA vasculitis and Kawasaki disease, exhibit distinct clinical phenotypes that seldom overlap with BD, making them less likely to be part of the differential diagnosis. Additionally, pediatric polyarteritis nodosa (PAN) is an exceedingly rare condition in children, and it is not included in our Registry data. Consequently, these were not added as control groups in our study. In light of these considerations, it is important to acknowledge that the actual accuracy of the classification criteria we analysed may be lower in real-world clinical practice. PEDBD criteria do not take into account the presence of disease clusters. In fact, it is well known that not all BD patients develop all the BD-related manifestations, but tend to present them in clusters (vascular; ocular-CNS; muco-cutaneous; papulo-pustules-arthritis-enthesitis) [4, 39–41]. Furthermore, important geographic and ethnic differences may play a role in the development of different manifestations. This may make the classification difficult, as not all patients fit the current classification criteria. Lastly, PEDBD are classification and not diagnostic criteria and should be therefore used to homogeneously classify BD patients for clinical trials and studies. Therefore, it is reasonable that some Eurofever patients enrolled and diagnosed as BD, were then not classified as BD according to these classification criteria.

In conclusion, this international effort was made to have a validated cohort of BD patients to which classification criteria could be applied to validate accuracy. The PEDBD criteria performed well, with very high specificity, but with lower sensitivity. The complexity of childhood BD suggests that genotyping (incorporating autoinflammatory diseases, BD mimics and HLA-type) combined with clinical features, disease clusters and ethnic variables are likely to ultimately yield the most accurate classification criteria, that would require further validation in larger prospective international cohorts.

## Supplementary Material

kead609_Supplementary_Data

## Data Availability

The data underlying this article are all available in the article and in its online supplementary material.
