# Correction to: Validation of the PEDiatric Behçet’s Disease classification criteria: an evidence-based approach

**DOI:** 10.1093/rheumatology/keae147

**Published:** 2024-03-18

This is a correction to: Caterina Matucci-Cerinic, Helene Palluy, Sulaiman M Al-Mayouf, Paul A Brogan, Luca Cantarini, Ahmet Gul, Ozgur Kasapcopur, Jasmin Kuemmerle-Deschner, Seza Ozen, David Saadoun, Farhad Shahram, Francesca Bovis, Eugenia Mosci, Nicolino Ruperto, Marco Gattorno, Isabelle Kone-Paut, on behalf of Eurofever for the Paediatric Rheumatology International Trials Organisation (PRINTO), Validation of the PEDiatric Behçet’s Disease classification criteria: an evidence-based approach, *Rheumatology*, 2023; kead609, https://doi.org/10.1093/rheumatology/kead609

In the originally published version of this article, the third author was incorrectly listed as Sulaiman Al-Mayouf. The article has been amended to read Sulaiman M. Al-Mayouf.

