# Supplementary material for: Validation of the PEDiatric Behçet’s Disease classification criteria: an evidence-based approach
Source: Rheumatology (Oxford). 2023 Nov 22;63(12):3422–31. doi: 10.1093/rheumatology/kead609 (PMC11636558; doi:10.1093/rheumatology/kead609)
Supplement: kead609_Supplementary_Data [file kead609_supplementary_data.zip › kead609_Supplementary_Data/rhe-23-1466-File008.docx]

**Supplementary Data**

The Author's group commissioned the study to the Paediatric Rheumatology International Trials Organisation which took care of the logistics of data collection/analysis and methodological support. The PRINTO network currently group 93 countries and more than 2000 members worldwide.

**Supplementary Table S1.** ISG, ICBD and PEDBD criteria

| **ISG criteria (8)** | **Revised ICBD criteria (10)** | **PEDBD criteria (5)** |
| --- | --- | --- |
| Oral Ulcers (> 3/year)  Plus 2 of:  - Genital Ulcers  - Eye lesions  Anterior uveitis  Posterior uveitis  Retinal vasculitis  - Skin lesions  Erythema nodosum  Pseudofolliculitis  Papulo-pustular lesions  Acneiform nodules  - Positive Pathergy Test | - Oral ulcers 2  - Genital ulcers 2  - Eye lesions 2  Anterior uveitis  Posterior uveitis  Retinal vasculitis  - Skin lesions 1  Erythema nodosum  Pseudofolliculitis  Skin ulcers  - Neurological manifestations 1  - Vascular manifestations 1  Venous thrombosis  Arterial thrombosis  Superficial Thrombophlebitis  -Positive Pathergy Test (additional) +1 | **-** Oral ulcers (≥3/year) 1  - Genital ulcers 1  - Eye lesions 1  Anterior uveitis  Posterior uveitis  Retinal vasculitis  - Skin lesions 1  Necrotic folliculitis  Acneiform lesions  Erythema nodosum  - Neurological manifestations 1  - Vascular manifestations 1  Venous thrombosis  Arterial thrombosis  Arterial aneurysms |
| **Classification: Oral ulcers + 2** | **Classification ≥ 4 points** | **Classification ≥ 3 points** |

**Supplementary Table S2.** Demographic characteristics of the initial cohort of 210 patients

| n° | **BD**  **70** | **FMF**  **35** | **MKD**  **26** | **TRAPS**  **22** | **PFAPA**  **40** | **UND/SURF 17** |
| --- | --- | --- | --- | --- | --- | --- |
| Gender, male/female (n) | 42/28 | 21/14 | 14/12 | 14/8 | 19/21 | 6/11 |
| Caucasian n (%) (European) | 67 (95.7) | 29 (82.8) | 25 (96.1) | 21 (95.4) | 35 (87.5) | 15 (88.2) |
| Middle-East n (%) | 1(1.4) | 3 (8.6) | 1 (3.8) | 1 (4.5) | 1(2.5) | 1 (5.9) |
| African n (%) | 1 (1.4) | 2 (5.7) | 0 | 0 | 0 | 1 (5.9) |
| Asian n (%) | 1 (1.4) | 0 | 0 | 0 | 1(2.5) | 0 |
| Hispanic (USA) n (%) | 0 | 1 (2.8) | 0 | 0 | 1(2.5) | 0 |
| Affected relatives n (%) | 12 (17.1) | 11 (3.1) | 1 (3.8) | 11(5.0) | 4(10) | 3(17.6) |
| Consanguinity n (%) | 1 (1.4) | 2 (5.7) | 0 | 1 (4.5) | 1(2.5) | 0 |
| Age at onset (years) median [1^st^-3^rd^ quartile] | 7.9  [4.1-11.8] | 1.9 [0.9-4.0] | 0.3 [0.1-0.7] | 2.0 [0.6-5.2] | 1.83 [1.0-2.9] | 1.0 [0.35-4] |
| Age at diagnosis (years) median [1^st^-3^rd^ quartile] | 10.4 [8.3-12.6] | 5.02 [3.4-6.7] | 4.4 [2.2-5.9] | 6.5 [2.9-7.9] | 3.5 [2.1-5.7] | 5.2 [3.1-10.5] |

BD, Behçet’s disease; FMF Familial Mediterranean Fever; MKD Mevalonate Kinase Deficiency; TRAPS TNF-Receptor Associated Periodic fever Syndrome; PFAPA Periodic Fever, Aphthous stomatitis, Pharyngitis, Adenitis; UND/SURF Undefined inflammatory syndromes/Syndrome of Undifferentiated Recurrent Fevers

**Supplementary Table S3.** Clinical characteristics of confirmed, probable, uncertain BD and of the initial BD with a different final diagnosis.

|  | **C-BD** | **P-BD** | **U-BD** | **DFD** |
| --- | --- | --- | --- | --- |
| N° patients | 24 | 10 | 7 | 29 |
| Male gender, n (%) | 16 (67) | 8 (80) | 4 (57) | 14 (48) |
| Age at onset (years) median [1^st^-3^rd^ quartile] | 7.95 [6.59-11.72] | 10.43 [6.55-11.97] | 7.9 [3.2-8.4] | 6 [3.1-9.8] |
| Age at diagnosis (years) median [1^st^-3^rd^ quartile] | 11.25 [8.48-12.31] | 11.85 [9.98-13.91] | 8.3 [8.2-8.7] | 10.2 [6.2-12.6] |
| Ethnicity, n (%) |  |  |  |  |
| Caucasian (European) | 23 (96) | 9 (90) | 7 (100) | 28 (97) |
| Asian | 1 (4) | 0 | 0 | 0 |
| Middle-East | 0 | 0 | 0 | 1 (3) |
| Other | 0 | 1 (10) | 0 | 0 |
| Affected relatives, n (%) | 4/23 (17) | 1 (10) | 0 | 7 (24) |
| Consanguineity, n (%) | 1 (4) | 0 | 0 | 0 |
| Clinical manifestation, n (%) |  |  |  |  |
| Maculopapular rash * | 1/23 (4) | 2 (20) | 1 (14) | 2 (7) |
| Urticarial rash | 0 | 0 | 1 (14) | 2 (7) |
| Erysipela-like rash | 1/23 (4) | 1(10) | 1 (14) | 0 |
| Migratory rash * | 0 | 0 | 1/6 (17) | 0 |
| Eritema polimorfo | 1 (4) | 0 | 0 | 0 |
| Malleolar ulcer | 0 | 1 (10) | 0 | 0 |
| erythematous plaques | 0 | 0 | 1 (14) | 0 |
| Psoriasis | 0 | 0 | 0 | 1 (3) |
| Palpable purpura * | 0 | 0 | 1 (14) | 0 |
| Ocular manifestations | 13 (54) | 2 (20) | 2 (29) | 7 (24) |
| Conjunctivits | 2/23 (9) | 0 | 2 (29) | 5 (17) |
| Anterior uveitis | 7 (29) | 1 (10) | 1 (14) | 5 (17 |
| Posterior uveitis * | 6/22 (27) | 0 | 1 (14) | 0 |
| Retinal vasculitis | 2 (8) | 0 | 0 | 0 |
| Papillary oedema | 2/23 (9) | 0 | 0 | 2 (7) |
| Papillitis | 1/23 (4) | 0 | 0 | 1 (3) |
| Impaired vision | 4 (17) | 0 | 1 (14) | 0 |
| Cataract | 1 (4) | 0 | 0 | 0 |
| Episcleriitis | 0 | 1 (10) | 1 (14) | 0 |
| Periorbital oedema | 0 | 0 | 0 | 1 (3) |
| Diplopia | 1 (4) | 0 | 0 | 0 |
| Vascular manifestations | 2 (8) | 0 | 0 | 1 (3) |
| Venous thrombosis | 2 (8) | 0 | 0 | 1 (3) |
| Neurologic manifestations | 10 (42) | 1 (10) | 1 (14) | 6 (21) |
| Isolated Headache | 6 (25) | 1 (10) | 1 (14) | 3 (10) |
| Headache any | 8 (33) | 1 (10) | 1 (14) | 5 (17) |
| Optic neuritis | 0 | 0 | 0 | 0 |
| Cranic nerves palsy | 4 (17) | 0 | 0 | 0 |
| Aspetic meningitis | 1 (4) | 0 | 0 | 0 |
| Vertigo | 2 (8) | 0 | 0 | 1 (3) |
| Behaviour abnormalities | 1 (4) | 0 | 0 | 0 |
| Neurosensorial hearing loss | 0 | 0 | 0 | 1 (3) |
| Mental retardation | 1 (4) | 0 | 0 | 1 (3) |
| Gastroenteric symptoms | 9 (38) | 3 (30) | 2 (29) | 11 (38) |
| Abdominal pain | 8 (33) | 3 (30) | 2 (29) | 11 (38) |
| Diarrhoea | 3 (13) | 0 | 1 (14) | 6 (21) |
| Constipation | 2 (8) | 0 | 0 | 0 |
| Gastrointestinal ulcers | 1 (4) | 0 | 0 | 0 |
| Anal/perianal ulcers | 2 (8) | 0 | 0 | 0 |
| GI bleeding | 3 (13) | 0 | 0 | 0 |
| Vomiting | 0 | 1 (10) | 0 | 1 (3) |
| Aspetic peritonitis | 0 | 1 (10) | 0 | 0 |
| Nephritis | 0 | 1 (10) | 0 | 0 |
| Musculo-skeletal manifestation | 8 (33) | 4 (40) | 6 (86) | 15 (52) |
| Arthralgia | 7 (29) | 4 (40) | 6 (86) | 14 (48) |
| Myalgia | 1/22 (4) | 1 (10) | 2 (29) | 9 (31) |
| Arthritis | 3 (13) | 3 (30) | 2 (29) | 3 (10) |
| Mono Arthritis | 1 (4) | 1 (10) | 0 | 0 |
| Oligoarthritis | 1 (4) | 0 | 1 (14) | 0 |
| polyarthritis | 1 (4) | 2 (20) | 1 (14) | 3 (10) |
| Bone pain | 0 | 1 (10) | 1 (14) | 0 |
| Tenosynovitis | 0 | 0 | 0 | 1 (3) |
| Bone alterations§ | 0 | 0 | 0 | 1 (3) |
| Generalized LN enlargement | 1 (4) | 1 (10) | 0 | 0 |
| Laterocervical LN enlargement | 0 | 0 | 2 (29) | 3 (10) |
| Erythematous pharyngitis | 1 (4) | 0 | 0 | 2 (7) |
| Exudative pharyngitis | 0 | 2 (20) | 2 (29) | 3 (10) |
| Pericarditis | 1 (4) | 0 | 0 | 0 |
| Fever * | 12 (50) | 2 (20) | 6 (86) | 8 (28) |
| regular periodic | 2 (8) | 1 (10) | 1 (14) | 3 (10) |
| irregular non periodic | 8 (33) | 1 (10) | 5 (71) | 5 (17) |
| Low fever | 4 (17) | 2 (20) | 3 (43) | 4 (14) |
| DC recurrent | 17 (71) | 8 (80) | 6 (86) | 23 (79) |
| DC continuous & recurrent | 7 (29) | 2 (20) | 1 (14) | 6 (21) |
| Fatigue | 4 (17) | 2 (20) | 2 (29) | 5 (17) |
| HLAB51 | 11/16 (69) | 5/7 (71) | 1/2 (50) | 11/19 (58) |

***Heterogeneity test*** * p < 0.05; BE bone erosions, C-BD Confirmed Behçet’s Disease; P-BD Probable BD, U-BD Uncertain BD; DFD initial BD patients with a different final diagnosis; Cd camptodactily, DC disease course, § Bone alterations : this patient presented withdigital clubbing, frontal bossing, Flexion contractures, hyperostosis, osteolytic lesions, patellar overgrowth, osteoporosis) LN lymphnodes,

**Supplementary Table S4.** Clinical features of the patients with confirmed-BD and confounding diseases

|  | **C- BD** | **TRAPS** | **MKD** | **FMF** | **PFAPA** | **UND/**  **SURF** |
| --- | --- | --- | --- | --- | --- | --- |
| N° patients | 24 | 17 | 24 | 17 | 26 | 31 |
| Aphtous stomatitis*(%) | 24 (100) | 1 (6) | 14 (58) | 1 (6) | 17 (65) | 17 (55) |
| Genital ulcers*(%) | 17 (71) | 0 | 1 (4) | 0 | 0 | 1 (3) |
| Pseudo-folliculitis(%) | 8 (33) | 1 (6) | 0 | 0 | 0 | 3 (10) |
| Papulo-pustular lesions^(%) | 7 (29) | 0 | 3 (13) | 0 | 0 | 2 (6) |
| Acne*(%) | 7 (29) | 0 | 0 | 0 | 0 | 2 (6) |
| Erythema nodosum(%) | 2 (8) | 0 | 0 | 0 | 0 | 0 |
| Positive Pathergy test*(%) | 7 (29) | 0 | 0 | 0 | 0 | 0 |
| Maculopapular rash^(%) | 1 (4) | 6 (35) | 7 (30) | 1 (6) | 0 | 3 (10) |
| Urticarial rash (%) | 0 | 2 (12) | 5 (22) | 1 (6) | 0 | 3 (10) |
| Erysipela-like rash (%) | 1 (4) | 2 (12) | 0 | 1 (6) | 0 | 0 |
| Migratory rash (%) | 0 | 3 (18) | 2 (9) | 1 (6) | 0 | 1 (3) |
| Erythema polymorfo (%) | 1 (4) | 0 | 0 | 0 | 0 | 0 |
| Malleolar ulcer (%) | 0 | 0 | 0 | 0 | 0 | 0 |
| Erythematous plaques(%) | 0 | 0 | 0 | 0 | 0 | 0 |
| Psoriasis (%) | 0 | 0 | 0 | 0 | 0 | 1 (3) |
| Palpable purpura (%) | 0 | 1 (6) | 1 (4) | 1 (6) | 0 | 1 (3) |
| Eye swelling (%) | 0 | 1 (6) | 0 | 0 | 0 | 0 |
| Feet oedema (%) | 0 | 1 (6) | 0 | 1 | 0 | 0 |
| Perioral eczema (%) | 0 | 0 | 1 (4) | 0 | 0 | 0 |
| Ocular manifestations(%) | 13 (54) | 7 (41) | 8 (35) | 0 | 1 (4) | 4 (13) |
| Conjunctivits^(%) | 2 (8) | 5 | 7 (30) | 0 | 1 (4) | 1 (3) |
| Anterior uveitis*(%) | 7 (29) | 0 | 0 | 0 | 0 | 3 (10) |
| Posterior uveitis*(%) | 6 (25) | 0 | 0 | 0 | 0 | 0 |
| Retinal vasculitis(%) | 2 (8) | 0 | 1 (4) | 0 | 0 | 0 |
| Papillary oedema(%) | 2 (8) | 0 | 0 | 0 | 0 | 0 |
| Papillitis(%) | 2 (8) | 0 | 0 | 0 | 0 | 1 (3) |
| Impaired vision(%) | 4 (17) | 1 (6) | 0 | 0 | 0 | 1 (3) |
| Cataract(%) | 1 (4) | 0 | 1 (4) | 0 | 0 | 0 |
| Episcleriitis(%) | 0 | 0 | 0 | 0 | 0 | 0 |
| Periorbital oedema(%) | 0 | 2 (12) | 0 | 0 | 0 | 0 |
| Periorbital pain^(%) | 0 | 0 | 0 | 0 | 3 | 1 |
| Corneal leukoma(%) | 0 | 0 | 0 | 0 | 0 | 1 (3) |
| Venous thrombosis(%) | 2 (8) | 0 | 0 | 0 | 0 | 0 |
| Neurologic manifestations(%) | 10 (42) | 0 | 11 (50) | 0 | 6 (23) | 8 (26) |
| Isolated Headache(%) | 6 (25) | 5 (29) | 7 (30) | 4 (24) | 6 (23) | 6 (19) |
| Cranic nerves palsy^(%) | 4 (17) | 0 | 1 (4) | 0 | 0 | 0 |
| Peripheral neuropathy(%) | 0 | 0 | 1 (4) | 0 | 0 | 0 |
| Diplopia(%) | 1 (4) | 0 | 0 | 0 | 0 | 0 |
| Aspetic meningitis(%) | 1 (4) | 0 | 0 | 0 | 0 | 0 |
| Vertigo(%) | 2 (8) | 0 | 0 | 0 | 0 | 0 |
| Behaviour abnormalities(%) | 1 (4) | 0 | 0 | 0 | 0 | 0 |
| Neurosensorial hearing loss(%) | 0 | 0 | 0 | 0 | 0 | 1 (3) |
| Mental retardation(%) | 0 | 0 | 1 (4) | 0 | 0 | 1 (3) |
| Seizures(%) | 0 | 0 | 2 (9) | 0 | 0 | 0 |
| Attention deficit | 0 | 0 | 0 | 0 | 0 | 1 (3) |
| Gastroenteric symptoms(%) | 9 (38) | 13 (76) | 23 (96) | 16 (94) | 9 (35) | 15 (48) |
| Abdominal pain*(%) | 8 (33) | 12 (71) | 23 (96) | 16 (94) | 9 (35) | 13 (42) |
| Diarrhoea*(%) | 3 (13) | 5 (29) | 20 (87) | 3 (18) | 0 | 9 (29) |
| Constipation (%) | 2 (8) | 1 (6) | 1 (4) | 1 (6) | 0 | 0 |
| Gastrointestinal ulcers(%) | 1 (4) | 0 | 2 (9) | 0 | 0 | 0 |
| Anal/perianal ulcers(%) | 2 (8) | 0 | 0 | 0 | 0 | 0 |
| GI bleeding(%) | 3 (13) | 0 | 3 (13) | 0 | 0 | 2 (6) |
| Vomiting*(%) | 0 | 4 (24) | 13 (57) | 3 (18) | 2 (8) | 2 (6) |
| Aspetic peritonitis(%) | 0 | 0 | 0 | 1 (6) | 0 | 0 |
| Intestinal occlusion/subocclusion(%) | 0 | 1 (6) | 0 | 0 | 0 | 0 |
| Nephritis(%) | 0 | 0 | 0 | 0 | 0 | 0 |
| Scrotal oedema(%) | 0 | 1 (6) | 0 | 0 | 0 | 0 |
| Epididymitis(%) | 0 | 0 | 0 | 1 (6) | 0 | 0 |
| Musculo-skeletal manifestations(%) | 8 (33) | 13 (76) | 17 (74) | 11 (65) | 5 (19) | 18 (58) |
| Arthralgia^(%) | 7 (29) | 11 (65) | 15 (65) | 10 (59) | 4/25 (16) | 13 (42) |
| Myalgia*(%) | 1 (4) | 11 (65) | 9 (39) | 10 (59) | 2/24 (8) | 8 (26) |
| Myositis, fascitiis(%) | 0 | 0 | 0 | 1 (6) | 0 | 1 (3) |
| Arthritis(%) | 3 (13) | 4 (24) | 1 (4) | 1 (6) | 0 | 4 (13) |
| Mono Arthritis(%) | 1 (4) | 2 (12) | 1 (4) | 0 | 0 | 0 |
| Oligoarthritis(%) | 1 (4) | 1 (6) | 0 | 1 (6) | 0 | 1 (3) |
| Polyarthritis(%) | 1 (4) | 1 (6) | 0 | 0 | 0 | 4 (13) |
| Bone pain(%) | 0 | 1 (6) | 0 | 1 (6) | 0 | 1 (3) |
| Tenosynovitis(%) | 0 | 0 | 0 | 1 (6) | 0 | 0 |
| Flexion contractures(%) | 0 | 0 | 0 | 0 | 0 | 1 (3) |
| BE/D/OL/H(%) | 0 | 0 | 0 | 0 | 0 | 1 (3) |
| Osteitis(%) | 0 | 0 | 0 | 0 | 0 | 1 (3) |
| Generalized LN enlargement ^(%) | 1 (4) | 5 (29) | 5 (22) | 0 | 1 (5) | 2 (6) |
| LC LN only*(%) | 0 | 4 (24) | 14 (61) | 3 (18) | 23 (88) | 9 (29) |
| Splenomegaly ^(%) | 0 | 2 (12) | 6 (26) | 1 (6) | 0 | 5 (16) |
| Hepatomegaly ^(%) | 0 | 0 | 5 (22) | 1 (6) | 0 | 2 (6) |
| Parotitis(%) | 0 | 0 | 0 | 0 | 0 | 1 (3) |
| Erythematous pharyngitis*(%) | 1 (4) | 3 (18) | 8 (33) | 2 (12) | 22 (85) | 7 (23) |
| Exudative pharyngitis*(%) | 0 | 0 | 6 (25) | 0 | 23 (88) | 6 (19) |
| Pericarditis(%) | 1 (4) | 2 (12) | 0 | 0 | 0 | 1 (3) |
| Chest pain*(%) | 0 | 2 (12) | 2 (9) | 7 (41) | 0 | 1 (3) |
| Pleurisy^(%) | 0 | 0 | 0 | 2 (12) | 0 | 2 (6) |
| Pneumonia(%) | 0 | 0 | 1 (4) | 1 (6) | 0 | 2 (6) |
| Persistent cough(%) | 0 | 0 | 0 | 2 (12) | 0 | 3 (10) |
| Hemoptysis(%) | 0 | 0 | 0 | 0 | 0 | 1 (3) |
| Fever*(%) | 12 (50) | 16 (94) | 24 (100) | 16 (94) | 26 (100) | 16 (52) |
| Regular periodic(%) | 2 (8) | 5 (29) | 7 (29) | 9 (53) | 20 (77) | 7 (23) |
| Irregular non periodic(%) | 8 (33) | 11 (65) | 17 (74) | 8 (47) | 6 (23) | 22 (71) |
| Low fever^(%) | 4 (17) | 1 (6) | 6 (26) | 0 | 2 (9) | 7 (23) |
| DC recurrent(%) | 17 (71) | 16 (94) | 22 (96) | 17 (100) | 25 (96) | 23 (74) |
| DC continuous & recurrent(%) | 7 (29) | 1 (6) | 1 (4) | 0 | 1 (4) | 8 (26) |
| Fatigue(%) | 4 (17) | 10 (59) | 11 (48) | 7 (41) | 6 (23) | 8 (26) |

***Heterogeneity test*** * p<0.001, ^p<0.05, C-BD confirmed-Behçet Disease; FMF Familial Mediterranean Fever; MKD Mevalonate Kinase Deficiency; TRAPS TNF-Receptor Associated Periodic fever Syndrome; PFAPA Periodic Fever, Aphthous stomatitis, Pharyngitis, Adenitis; UND/SURF Undefined inflammatory syndromes/Syndrome of Undifferentiated Recurrent Fevers; LN lymphnodes, LC laterocervical

**Supplementary Table S5.** Genetic characteristics of the patients with monogenic autoinflammatory diseases

| FMF (MEFV, NM_001243133.1) | | | MKD (MVK, NM_000431.3) | | | TRAPS (TNFRSF1A, NM_001065.3) | |
| --- | --- | --- | --- | --- | --- | --- | --- |
| **Mutation 1** | **Mutation 2** | **N° pts** | **Mutation 1** | **Mutation 2** | **N° pts** | **Mutation** | **N° pts** |
| M694V | M694V | 3 | V377I | V377I | 3 | R92Q | 11 |
| M694V | E230K | 1 | V377I | I268T | 3 | N65I | 2 |
| M680IGC | nt 2177 T>C | 1 | V321A | N205D /S52N | 1 | T50M | 3 |
| K695R | M694V/ E148Q | 1 | V377I | L264F | 2 | C55Y | 1 |
| M680IGC | M680IGC | 1 | V377I | G336S | 1 | C88Y | 1 |
| M680IGA | R761H | 1 | V377I | C152Y | 1 | C33G | 1 |
| M680IGA | M680IGA | 1 | V377I | V310M | 1 | C52Y | 1 |
| R761H | E148Q | 1 | G336S | R215Q | 1 | T61N | 1 |
| V726A | S108R | 1 | V377I | L265R | 1 |  |  |
| M694DEL | R202Q | 1 | V377I | P165L | 1 |  |  |
| V726A | S108R | 1 | V377I | V203fs*74 | 2 (1G, 1S) |  |  |
| M694V | V726A | 1 | V377I | R215Q | 2 |  |  |
| M694V | E148Q | 1 |  |  |  |  |  |
| E148Q | E148Q | 1 |  |  |  |  |  |
| M680IGC | V726A | 1 |  |  |  |  |  |
|  |  |  |  |  |  |  |  |
| M694V |  | 6 | V377I |  | 4 |  |  |
| E148Q |  | 3 | I268T |  | 2 |  |  |
| M694I |  | 1 | I268T |  | 1 |  |  |
| K695R |  | 1 | R215Q |  | 1 |  |  |
| E148Q |  | 1 |  |  |  |  |  |
| V726A |  | 1 |  |  |  |  |  |
| G219G |  | 1 |  |  |  |  |  |
| F479L |  | 1 |  |  |  |  |  |
| V726A |  | 1 |  |  |  |  |  |
| R202Q |  | 1 |  |  |  |  |  |
| Not done |  | 2 |  |  |  | Negative | 1 |
|  |  |  |  |  |  |  |  |
| **Other mutations** | Associated to |  | **Other mutations** | Associated to |  |  |  |
| TNFRSF1A S726G | hetG219G | 1 | NLRP3 V198M | hetV377I |  |  |  |
|  |  |  | MEFV V310M | het V377I |  |  |  |
|  |  |  | MEFV R202Q | V377I/ V203Sfs and V377I/L264F |  |  |  |

FMF Familial Mediterranean Fever; MKD Mevalonate Kinase Deficiency; TRAPS Tumor necrosis factor (TNF) receptor-associated periodic fever syndrome; het heterozygous

**Supplementary Table S6.** Genetic characteristics of the patients with polygenic autoinflammatory diseases

| **Gene** | **UND/SURF (17)** | | **PFAPA** **(40)** |  |
| --- | --- | --- | --- | --- |
|  | Mutation | N° pts | Mutation | N° pts |
| **MEFV** | hetK695R | 1* | E148Q | 1 |
| **TNFRSF1A** | R92Q | 1* | R92Q | 1 |
| **Negative** |  | 9 |  | 14 |
| **Not done** |  | 7 |  | 24 |

* Same patient; PFAPA Periodic Fever, Aphthous stomatitis, Pharyngitis, Adenitis; UND/SURF Undefined inflammatory syndromes/Syndrome of Undifferentiated Recurrent Fevers

**Supplementary Table S7. Fulfilment of the ISG, ICBD and PEDBD criteria in the whole cohort of patients with a consensus**

|  | **BD** | **FMF** | **MKD** | **TRAPS** | **PFAPA** | **UND/SURF** |
| --- | --- | --- | --- | --- | --- | --- |
| **ISG (%)** | 12 (50) | 0 | 0 | 0 | 0 | 0 |
| **ICBD (%)** | 19 (79.2) | 0 | 2 (8.3) | 0 | 0 | 1(3.6) |
| **PEDBD (%)** | 14 (58.3) | 0 | 1(4.2) | 0 | 0 | 0 |

Data are presented as frequencies (%). BD Behçet’s Disease; FMF Familial Mediterranean Fever; MKD Mevalonate Kinase Deficiency; TRAPS TNF-Receptor Associated Periodic fever Syndrome; PFAPA Periodic Fever, Aphthous stomatitis, Pharyngitis, Adenitis; UND/SURF Undefined inflammatory syndromes/Syndrome of Undifferentiated Recurrent Fevers
