# Supplementary figures and images for: Validation of the PEDiatric Behçet’s Disease classification criteria: an evidence-based approach
Source: Rheumatology (Oxford). 2023 Nov 22;63(12):3422–31. doi: 10.1093/rheumatology/kead609 (PMC11636558; doi:10.1093/rheumatology/kead609)

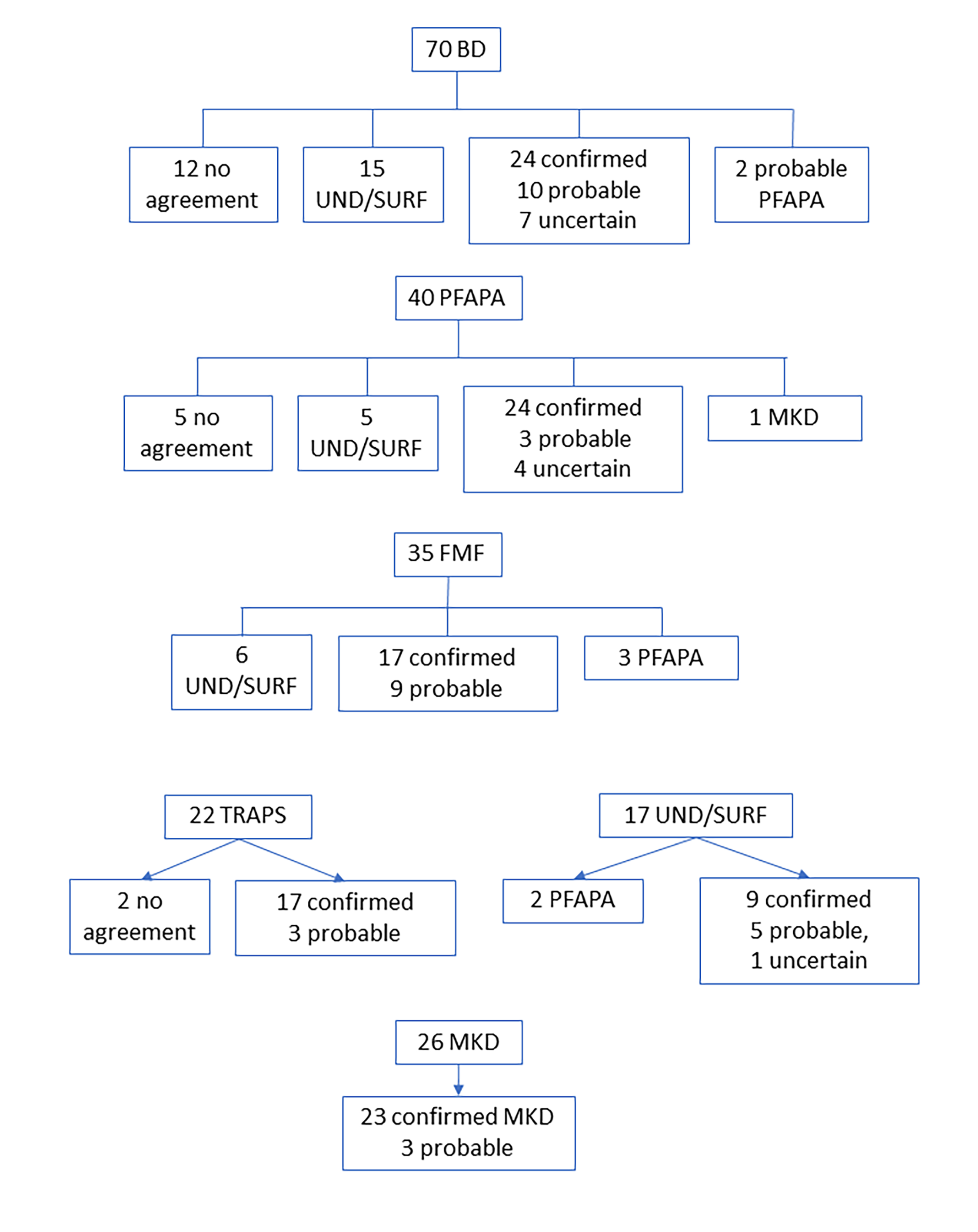

Supplement: kead609_Supplementary_Data [file kead609_supplementary_data.zip › kead609_Supplementary_Data/rhe-23-1466-File005.tif]

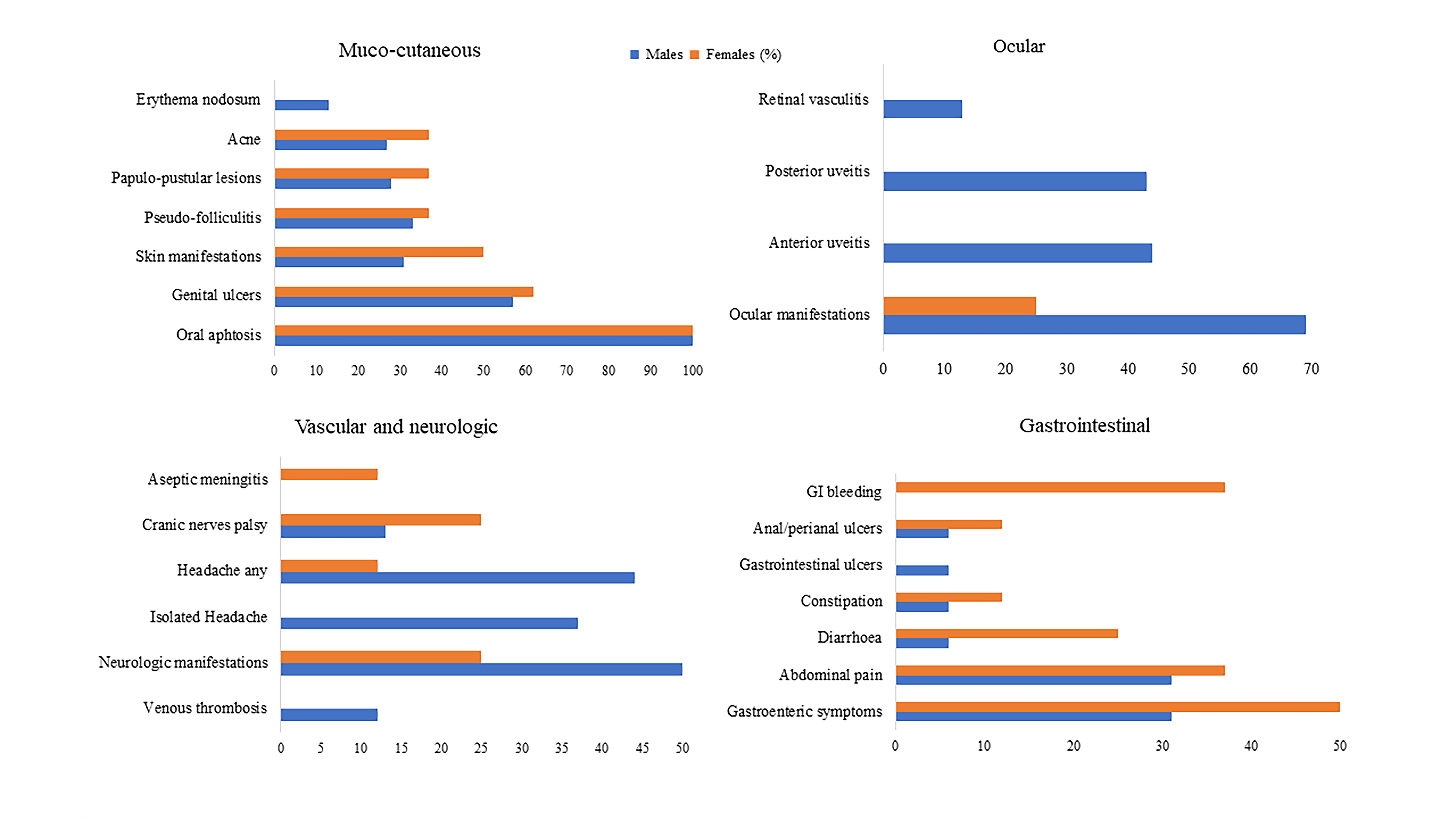

Supplement: kead609_Supplementary_Data [file kead609_supplementary_data.zip › kead609_Supplementary_Data/rhe-23-1466-File006.tif]

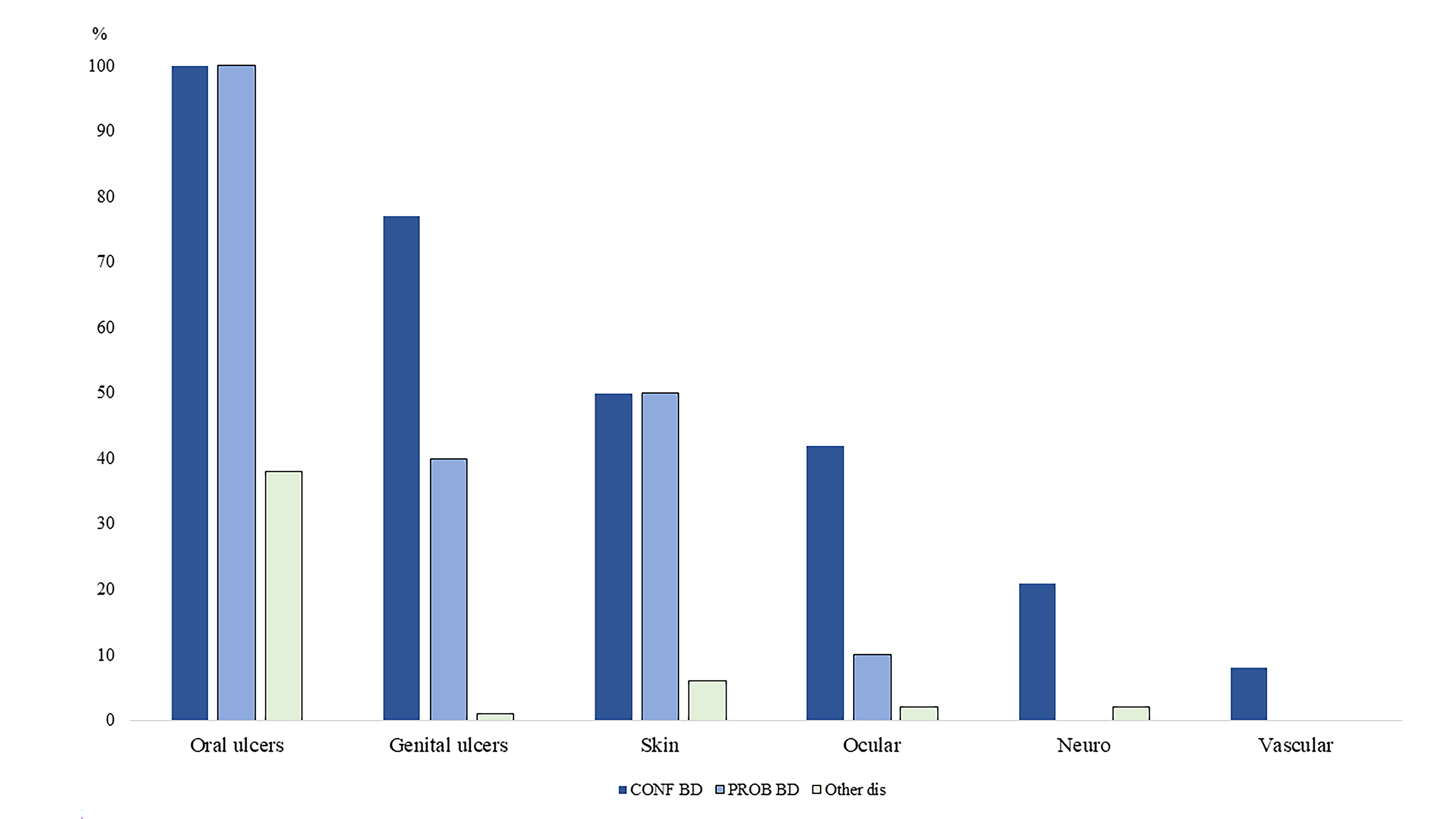

Supplement: kead609_Supplementary_Data [file kead609_supplementary_data.zip › kead609_Supplementary_Data/rhe-23-1466-File007.tif]
